# Cost analysis of school-based sexuality education programs in six countries

**DOI:** 10.1186/1478-7547-11-17

**Published:** 2013-08-01

**Authors:** Jari Kivela, Evert Ketting, Rob Baltussen

**Affiliations:** 1 Qalys Health Economics, Middenweg 239 I, 1098AP, Amsterdam, Netherlands; 2 Nijmegen International Center for Health Systems Research and Education (NICHE), Department of Primary and Community Care, Radboud University Nijmegen Medical Center, 9101, Nijmegen, 6500HB, Netherlands

**Keywords:** Sexuality education, Sexual health, School-based, Cost, Scale-up, HIV prevention

## Abstract

**Background:**

Policy-makers who are making decisions on sexuality education programs face important economic questions: what are the costs of developing sexuality education programs; and what are the costs of implementing and scaling them up? This study responds to these questions by assessing the costs of six school-based sexuality education programs (Nigeria, Kenya, Indonesia, India, Estonia and the Netherlands).

**Methods:**

Cost analyses were carried out in schools that were fully implementing a SE program, as this best reflects the resources needed to run an effective program. The costs were analyzed from the program perspective, meaning that all costs borne by the governmental and (international) non-governmental organizations supporting the program were included. Cost analyses were based on financial records, interviews and school surveys.

We distinguished costs in three consecutive program phases: development, update and implementation. Recommendations on the most efficient program characteristics and scale-up pathways were drawn from results of three fully scaled up programs (Estonia, Nigeria and the Netherlands), scale-up scenarios of two pilot programs (Kenya and Indonesia), and an implementation plan (India), The costs of the programs were compared by converting cost per student reached in US dollars (US$) to international dollars (I$).

**Results:**

Findings revealed a range of costs and coverage of sexuality education programs. Costs per student reached were; US$7 in Nigeria, US$13.50 in India, US$33 in Estonia and the Netherlands, US$50 in Kenya, and US$160 in Indonesia.

**Conclusions:**

Intra-curricular sexuality education programs have, because of their compulsory nature, the most potential to be scaled up and are therefore most efficient. Extra-curricular sexuality education programs have lower potential to be scaled up and are therefore less efficient. In terms of class size and number of lessons, countries need to strike a balance between the quality (demanding smaller classes and many lessons) and the costs (demanding larger classes and fewer lessons). Advocacy was a significant cost component.

## Background

Policy-makers worldwide who are involved in decisions about sexuality education programs face important economic questions: what are the costs of developing sexuality education programs; and what are the costs of implementing and scaling them up? Knowing the answers to these questions would enable policy-makers to invest resources in sexuality education more efficiently. This study responds to the above questions by assessing the costs of school-based sexuality education (SBSE) programs in six countries (Nigeria, Kenya, Indonesia, India, Estonia and the Netherlands) and program scale-up projections in two countries (Kenya and Indonesia).

This study fills in important gaps on the economic aspects of sexuality education programs worldwide, in low-, middle- and high-income countries. It also comes at a time when interest in sexuality education programs is growing considerably.

This article only considers costs of SBSE programs. Health effects of these SBSE programs are notoriously difficult to measure. As an integrated part of our study we made efforts to measure health effects of the Estonian program, which will be reported in a companion paper [1].

The cost estimates in this article are relevant not only to the countries and sexuality education programs studied, but also to other countries considering implementing or scaling up existing sexuality (including HIV prevention) education programs.

### Sexuality education programs analyzed

The selection of countries reflects a broad geographical spread, with two countries in Africa (Nigeria and Kenya), two in Asia (Indonesia and India), and two in Europe (Estonia and the Netherlands). It also reflects a range of experiences. The Netherlands has a fairly long tradition of sexuality education, whereas in Indonesia and Kenya, recently implemented pilot programs are evaluated, operating on a relatively small scale. Estonia introduced its program about 15 years ago, though it is only now firmly established nationwide. In Nigeria, the sexuality education program started in Lagos state, and is now also implemented in Abuja state. India is in the course of implementing a program in the Orissa State. Table 1 summarizes characteristics of the programs studied. Detailed program descriptions can be found elsewhere [2].

**Table 1:** Characteristics of school-based sexuality education programs

**Country**	**Nigeria**	**Kenya**	**Indonesia**	**India**	**Estonia**	**The Netherlands**
Name of SE program	Family Life and HIV Education	World Starts With Me	Daku!	Adolescent reproductive and sexual health curriculum	Human Studies (SE is part of it)	Long Live Love
Geographical area for program evaluation	Lagos State	4 provinces	4 provinces	Orissa State	Whole country	Whole country
Intra- / extra- curricular	Intra-curricular	Extra-curricular	Extra-curricular	Intra-curricular	Intra-curricular	Intra-curricular
Integrated / stand-alone	Integrated	Stand-alone	Stand-alone	Integrated	Integrated	Stand-alone
Targeted age-group	13-15 y	13 -16 y	15-17 y	13-16 y	7-14 y	13-15 y
Class	Junior secondary school (grades 1–3)	Secondary school (grades 1–4)	Senior high school (grade 2)	High school (grades 8–10)	Basic school (grades 1–7)	Secondary school(grade 2 or 3)
Program duration (years)	3	1	1	3	3(7)	1

The success of sexuality education programs is largely determined by the context in which they are developed and implemented, as well as by their characteristics and the quality of implementation. In many countries, sexuality, and therefore sexuality education, is a sensitive issue that may generate opposition. Where there is opposition, the introduction of sexuality education requires careful planning and a wide variety of advocacy and public education activities. This has a significant effect on the costs and impact of the programs (see below).

Among international experts there is a strong consensus that sexuality education programs that are fully integrated into the school curriculum are preferable to stand-alone programs. However, in many countries, the conditions for fully integrated sexuality education programs are not sufficient, and therefore extra-curricular, stand-alone programs are the only ones that may currently be possible. The programs in Indonesia and Kenya are of the latter type.

## Methods

This report presents the results of a comprehensive costing analysis – based on detailed inspection of financial records, interviews with sexuality education program personnel, and primary data collection through specifically designed surveys in schools – in all countries studied. Cost analyses were carried out in schools that were fully implementing a SE program, as this best reflects the resources needed to run an effective program. The exception is Orissa State, India, where program implementation began in 2010, and where the costing analysis is therefore estimated on the basis of implementation *plans*. More detailed costing methods and resource use and prices can be found elsewhere [2].

Analyses were conducted from the program perspective, including all costs as borne by governmental and (international) non-governmental organizations supporting the program. The economic costs of the sexuality education programs were estimated, including all resources used, and the budgetary outlays were also calculated, i.e. the costs of running the programs additional to already existing expenses for teacher salaries. Various program phases – development or adaptation, implementation (including program scale-up), and update – and attendant inputs were identified to reflect all resources required for developing and implementing a sexuality education program. Recommendations on the most efficient program characteristics and scale-up pathways were drawn from results of three fully scaled up programs (Estonia, Nigeria and the Netherlands), scale-up scenarios of two pilot programs (Kenya and Indonesia), and an implementation plan (India).

All costs were analyzed in local currencies and converted to US dollars (US$) using 2009 exchange rates [3]. The initial program development costs were considered as capital goods and annualized over ten years. Costs related to program adaptations, teacher trainings and computers (in Kenya and Indonesia) were annualized over five years [4]. To make meaningful comparisons of the costs of the sexuality education programs across the study countries, one indicator was chosen: cost per student reached in 2009. The costs per student for the duration of the entire curriculum were estimated, and thus accounted for differences in the length of sexuality education programs across countries. Costs per student reached in US dollars were converted to international dollars (I$) by using purchasing power parity indicators [5].

International dollars have the advantage that they account for the difference in price levels between countries, and allow for a comparison of the actual resource use by the sexuality education programs in the countries concerned.

The overall approach adhered to the WHO-CHOICE methodology on costing analysis – an internationally accepted standard for the conduct of economic analysis of health programs, especially in low- and middle-income countries [6].

## Results

Study findings reveal a wide range of costs and coverage of sexuality education programs across the countries studied. Total costs of sexuality education programs, including development or adaptation, updating and implementation, range between US$1.19 million in Indonesia to US$12.1 million in the Netherlands. The total number of students reached varies from some 6,000 in Indonesia, to 990,000 in India (as planned for the period 2010–2014). This is dependent on the scale of the program and the number of years it is implemented in the country, and therefore the report concentrates on annual figures. The annualized costs and the annual number of students reached in 2009 are US$562,000 and 246,000 students in Nigeria; US$364,000 and 7,300 students in Kenya; US$289,000 and 1,800 students in Indonesia; US$3.5 million and 780,000 students in India (as planned in 2014); US$311,000 and 28,000 students in Estonia; and US$830,000 and 25,300 students in the Netherlands. In every country, the majority of all costs are implementation (including scaling up) costs, and costs of program development, adaptation and updating are minor. Table 2 summarizes the main findings. Detailed analysis of development, adaptation, implementation and updated costs can be found elsewhere [7].

**Table 2:** Comparison of findings of the cost analysis (US$ 2009 prices)

**Country**	**Nigeria**	**Kenya**	**Indonesia**	**India [i]**	**Estonia**	**Netherlands [ii]**
Name of SE program	Family Life and HIV Education	World Starts With Me	Daku!	Adolescent reproductive and sexual health curriculum	Human Studies (SE is part of it)	Long Live Love
Period considered	1999–2009	2005–2009	2005–2009	2010–2014	1991–2009	1999–2009
Program duration (years) (a)	3	1	1	3	3 (7)	1
Total program costs (b)	3,400,000	1,380,000	1,200,000	10,800,000	5,610,000	12,200,000
Annualized costs in 2009 (c)	562,000	364,000	289,000	3,502,000	311,000	830,000
Schools covered in 2009 (d)	319	112	77	5,560	382	174
Average class size (students)	75 - 150	44	30	45	18	20
Average SE teaching time per class per year (hours)	14.2 hours per class per year 42.7 hours over three years	46.3 hours per class per year	47.2 hours per class per year	7.9 hours per class per year 23.7 hours over three years	33. hours over three years	11.4 hours per class (one LLL lesson is 2.2 hours)
Teachers’ monthly gross salary	350	467	276	298	1,500	4,137
Cost per school reached in 2009 (e) = (c)/(d)	1,762	3,250	3,750	630	814	4,768
Cumulative number of students reached (f)	694,000	13,000	6,240	990,000	190,000	376,000
Students covered in 2009 (g)	246,000	7,300	1,805	780,000	28,000	25,300
Cost per student reached in 2009 (h) = (c)/(g)	2.28	49.98	159.93	4.49	11	32.80
Cost per student reached (who completed the curriculum) in 2009 (i) = (a)*(h) [iii]	6.90	50	159.90	13.50	32.90	32.80
Budgetary outlays per student reached (who completed the curriculum) in 2009 [iv]	0.62	37.20	135.44	2.52	8.39	10.40

^i^India data refers to the planned implementation period 2010–2014. Where 2009 is stated, it should read 2014.

^ii^The Netherlands refers to program version LLL3 during the period 2001–2009. Earlier versions LLL1 and LLL2 during the period 1991–1998 were excluded from the study.

^iii^Cost per student reached refers to students who have completed the curriculum. The costs are calculated as the duration of the curriculum multiplied by the costs per student covered in 2009.

^iv^Budgetary outlays refers to MoE’s additional cost of providing sexuality education; costs that occur in addition to the regular expenses, e.g. teacher salaries that are paid also in the absence of a SBE program.

In the interpretation of the main findings – the cost per student reached across countries – it must be stressed from the outset that the sexuality education programs in Kenya and Indonesia are much more costly because they are still in a pilot phase and therefore small scale. Costs per student reached were US$7 in Nigeria and US$13.50 in India, US$33 in Estonia and the Netherlands, US$50 in Kenya, and US$160 in Indonesia.

**Figure 1:**
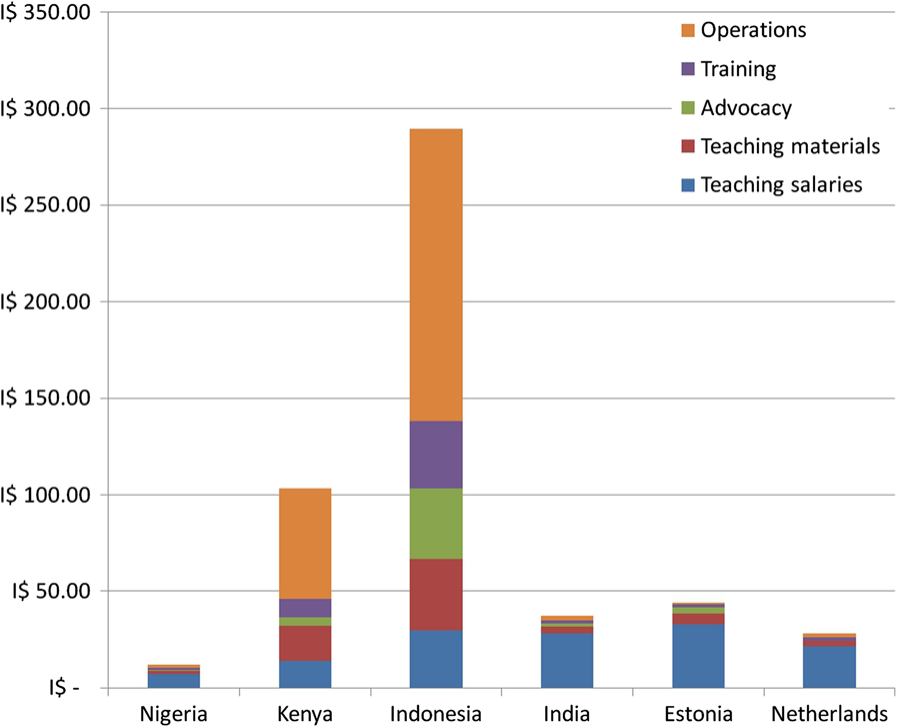
Sexuality education program cost per student reached by activity in international dollars (I$).

Figure 1 shows the distribution of implementation costs by activity, including teaching salaries. The costs per student are expressed in international dollars (I$) to make more meaningful comparisons. The programs in Kenya and Indonesia comprise relatively large operations costs per student reached, including personnel of the implementing NGOs, office and travel. In Nigeria, India, Estonia and the Netherlands, these costs are much lower, and the largest share of costs is teacher salaries. Training, advocacy and teaching material costs vary between countries, but each of these activities never account for more than 20 per cent of total costs in the analysis.

However, if only budgetary outlays are considered, i.e. the costs in addition to regular expenses on teacher salaries, costs per student reached fall to US$0.60 in Nigeria, US$2.50 in India, US$8 in Estonia, US$10 in the Netherlands, US$37 in Kenya and US$135 in Indonesia. In India, Estonia and the Netherlands, for example, these budgetary outlays constitute 0.5, 0.2 and 0.1 per cent, respectively, of current expenditure per student in secondary education.

Results of the scale-up scenarios of Kenyan and Indonesian programs suggest that the most efficient strategy is to first increase program uptake in the current schools, for example by making the curriculum mandatory, before introducing the program to new schools or districts. Moreover, in case these pilot programs would be scaled up to 300 schools and achieved (mandatory) full coverage, costs per learner reached would be reduced to US$16 in Kenya and US$13.40 in Indonesia.

## Discussion

### Sensitivity of sexuality education and its effect on costs

The sexuality education programs in Nigeria, Kenya, Indonesia and India have been implemented in contexts where sexuality, and therefore sexuality education, is a sensitive issue. In contrast, sexuality education is not a sensitive issue in Estonia or the Netherlands. The sensitivity of the topic has important consequences for how and the pace at which sexuality education programs can be introduced, their character (comprehensive versus abstinence-only), and the scale at which they can be carried out. This has an effect on costs and potential impact. In Nigeria and India, sexuality education programs initially came to a halt because of socio-cultural opposition, thereby causing years of delay and related loss of investments. In Nigeria, the initial comprehensive program had to be reduced: all elements related to actual sexual and preventive behavior, including contraception and condoms were removed. The programs in Orissa State, India (as planned) and Estonia are good examples of comprehensive, integrated and fully scaled up sexuality education programs, and these hold important lessons for other countries that wish to achieve similar scales and related impact. The programs in Kenya and Indonesia are NGO-initiated, also in response to the sensitivity of sexuality education and the relative hesitance of national governments to address the topic. These programs are extra-curricular, voluntary and seem to be constrained in the coverage they can achieve. However, they can be an important stepping stone toward the development of national sexuality education programs. All sexuality education programs in all countries require careful planning and a wide variety of advocacy and public education activities to achieve their implementation.

### Costs of sexuality education in relation to program design

The programs in Nigeria, India, Estonia and the Netherlands appear to be relatively inexpensive in terms of cost per student reached, costing approximately US$7, US$14, US$33 and US$33, respectively. These programs are all intra-curricular and implemented on a large scale (annually reaching 25,000 to 250,000 students), which reduces costs per student of national and state-level activities, such as program development, management and advocacy. Also important is the mandatory student enrolment in these programs, resulting in an almost comprehensive coverage of enrolled students per school. This reduces school-level costs per student, such as teachers’ salaries (in all programs, teacher salaries are a major cost component). On this basis, we conclude that intra-curricular sexuality education programs are most efficient, and we refer to the program in Estonia and the planned program in India as best examples in this respect. The sexuality education program in the Netherlands is difficult to interpret in this context, because the program is relatively short, and functions as an addition to a more elaborate sexuality education at primary school, and in biology lessons in secondary school.

The sexuality education programs in Kenya and Indonesia appear to be relatively costly, at US$50 and US$160 per student reached, respectively. These programs are currently in the pilot phase, geographically spread out, and initiated by international and national NGOs. At this stage, these programs have limited coverage – annually reaching between 1,800 and 7,300 students respectively – and carry high operations (salary and travel) costs. Cost per student would diminish considerably if the programs were scaled up beyond the pilot phase. However, both programs are also extra-curricular and thus voluntary, so the potential of such programs to achieve widespread coverage is questionable. Integration of the program in the regular curriculum would be a possible strategy to meet that concern. However, such programs may sometimes be the only available option in a country where sexuality education is a sensitive issue, and this could be a reason to accept their relatively high cost during a period of transition.

In addition, the sexuality education programs in Kenya and Indonesia are both computer-based, and this also makes them relatively costly because it necessarily reduces class size (schools have a limited number of computers). Uptake in schools – between 42 students per class in Indonesia and 44 students in Kenya – is constrained as a result. This hinders the scale-up of a program across and within schools, and its integration into the regular curriculum.

Teacher salaries are a major cost component in all programs, and class size strongly influences cost per student reached. In Nigeria, classes for the sexuality education lessons usually have 75 to 150 students, while classes are much smaller in, for example, India (around 40 students) and Estonia (around 18 students). While large classes are thus favorable for cost purposes, the quality of implementation in such classes will likely be compromised. Even when specific strategies are developed in sexuality education to cope with large classes, as in Nigeria, sexuality education typically requires interactive teaching methods with high levels of student involvement, which can hardly be realized in overcrowded classes.

Advocacy costs are a significant cost component in all countries, ranging between 4 per cent of total costs in Kenya to 13 per cent of total costs in Indonesia. The only exception was the Netherlands, where advocacy costs were 0.1 per cent. Therefore, advocacy costs seem to be highest where there is most resistance towards sexuality education. Advocacy costs are incurred not only in the development phase of the program but also throughout its implementation, and reflect the sensitive nature of sexuality education curricula in a country. Advocacy includes a broad scope of activities including political lobbying, media activities, stakeholder meetings, working groups, sensitization meetings for school staff, parents, and health care providers, and exhibitions.

Programs examined in this study differ in the way they were developed and/or adapted. Development costs in Estonia were low because of low salary levels during the first years of independence, and programs did not require intensive advocacy activities. Programs in Kenya and Indonesia were adapted from a similar program in Uganda and had important savings in the development costs of the original software. However, the adaptation process was still relatively costly as these computer-based programs required expensive software adaptation activities. Moreover, the adaptation was supported by an international organization, which added extra costs. The adaptation costs in these countries constituted between 15 per cent and 24 per cent of total costs. Development and updating costs ranged between 1 per cent and 11 per cent of total costs in the other study countries. Finally, the proportion of development or adaptation costs is also dependent on the number of years a program has been implemented in a country.

The duration of the programs varies. The number of learning hours per student over the duration of the curriculum varies between 11 hours in the Netherlands (which is additional to an extensive foundation established at the primary level and to biology classes), to some 40 hours in most other countries. Obviously, this is closely related to the cost per student reached. The number of learning hours also determines the impact of a program – international standards recommend at least 12 to 20 lessons (typically lasting 45 minutes to an hour) over several years.

### Budget impact of sexuality education programs

The budget impact of implementing sexuality education programs is not equal to the economic costs as presented in this study. Teacher salaries are included as economic costs in this study but are a regular expense of the Ministry of Education, irrespective of the implementation of sexuality education programs. In the implementation of a sexuality education program, these salaries would therefore not incur additional budgetary outlays. This study shows that budgetary outlays are less than 25 per cent of the economic costs of sexuality education programs (with the exception of Kenya and Indonesia) and, as estimated in this study, range from US$0.60 in Nigeria to US$10 in the Netherlands. However, it should be noted that the introduction of a sexuality education program in the curriculum could come at the expense of not teaching other courses, which is an opportunity cost.

### Efficient pathways to scaling up programs

In order to assess the cost implications of scaling up sexuality education programs, we defined several different scenarios and, based on these, we recommend the most efficient pathways to greater sexuality education coverage. The most efficient strategy appears to be to start first expanding program uptake in schools where the program is currently being implemented, for example, by making the curriculum mandatory, before introducing the program to new schools or districts. Again, this is because teacher salaries constitute a major cost component and are reduced by covering more students per class or school. The ideal strategy from the efficiency point of view is to maximize uptake in schools and the coverage of schools in a country.

### Limitations

A number of limitations were faced in conducting the study. First, information on the actual number of learners reached was not always available, especially in the larger intra-curricular programs. In those instances, program coverage was calculated on the basis of secondary sources, e.g. student materials used or teachers trained. Second, it is not always clear where sexuality education programs begin and end. Sexuality education programs are sometimes part of wider life-skills programs, as in Estonia. Since general life skills, such as decision-making competence, serve more purposes than only promoting healthy sexual behavior, it is then somewhat arbitrary to determine which part of the program should be labeled sexuality education. Third, it was not always possible to make detailed assessments of costs. Some programs, such as curricula in Estonia and the Netherlands, have existed for a long time, and financial records were not always available. In those instances, the analysis was based on gross estimates from program personnel.

This study only focuses on the costs of sexuality education programs. It does not answer several other very important questions, such as variation in the quality of different types of programs, nor does it address the important question of how to develop a sexuality education program and integrate it into existing curricula. This requires additional efforts beyond the scope of this study, which could result in a strategic document that outlines pathways, under different conditions, for successfully developing and integrating sexuality education in school curricula.

## Conclusions

On the basis of the above analysis we draw the following conclusions and recommendations.

Intra-curricular sexuality education programs have, because of their compulsory nature, the most potential to be scaled up – in terms of coverage of schools and students in schools – and are therefore most efficient. Where possible, we recommend this type of sexuality education program.

Extra-curricular sexuality education programs have, because of their voluntary nature, lower potential to be scaled up and are therefore less efficient. These programs are therefore not recommended. However, they can be important learning experiences and stepping stones to national sexuality education programs, or may be the only available option in a country considering the sensitive nature of sexuality education. Where possible, such programs should be gradually integrated in the national curriculum to render them more efficient.

In terms of class size and number of lessons, countries need to strike a balance between the quality (demanding relatively small class sizes and many lessons) and the costs (demanding relatively large class sizes) of sexuality education programs. Furthermore advocacy appeared to be a significant component of the costs of sexuality education programs in most countries, and we recommend that educational authorities consider this to be a necessary investment. Next we recommend that new initiatives save costs by adapting existing programs to their own (social and cultural) context, instead of developing new ones. We also recommend that sexuality education programs wishing to increase their coverage start by expanding program uptake among students in schools first (e.g. by making the curriculum mandatory), before introducing the program to new schools or districts. Finally where student access to computers is limited, computer-based sexuality education programs are not recommended.

## Competing interests

The authors declare that they have no competing interests.

## Authors’ contributions

JK is a health economist consultant. EK is a sexual and reproductive health & rights expert. RB is an associate professor at Radboud University Nijmegen Medical Center. EK, RB and JK designed the study. JK coordinated the data collection and performed the health economic analyses with RB. EK was responsible for the description and context of the six school-based sexuality education programs. JK, EK and RB wrote the manuscript. All authors read and approved the final manuscript.
